# Correction: Molecular architecture of the 90S small subunit pre-ribosome

**DOI:** 10.7554/eLife.29876

**Published:** 2017-06-26

**Authors:** Qi Sun, Xing Zhu, Jia Qi, Weidong An, Pengfei Lan, Dan Tan, Rongchang Chen, Bing Wang, Sanduo Zheng, Cheng Zhang, Xining Chen, Wei Zhang, Jing Chen, Meng-Qiu Dong, Keqiong Ye

Sun Q, Zhu X, Qi J, An W, Lan P, Tan D, Chen R, Wang B, Zheng S, Zhang C, Chen X, Zhang W, Chen J, Dong M-Q, Ye K. 2017. Molecular architecture of the 90S small subunit pre-ribosome. *eLife* **6**:e22086. doi: 10.7554/eLife.22086.

Published 28, February 2017

In the published article, two typos have been corrected.

In the second sentence of the third paragraph of the chapter "UTPA", the text "The crystal structures of the N-terminal region (N domain) of Utp10 in complex with a C-terminal peptide of Utp17 and a middle region (M domain) of Utp10 were docked into two separated places in the density (PDB codes 2WYL, 5WY3)" has been replaced with "The crystal structures of the N-terminal region (N domain) of Utp10 in complex with a C-terminal peptide of Utp17 and a middle region (M domain) of Utp10 were docked into two separated places in the density (PDB codes 5WYL, 5WY3)".

In the second sentence of the fourth paragraph of the chapter "The central domain", the text "The density, although weak, was well fitted by the crystal structure of the C-terminal tetratricopeptide repeat (TPR) domain of Rrp5 (PDB code 3WWM and [Khoshnevis et al., 2016])" has been replaced with "The density, although weak, was well fitted by the crystal structure of the C-terminal tetratricopeptide repeat (TPR) domain of Rrp5 (PDB code 5WWM and [Khoshnevis et al., 2016])".

The article has been corrected accordingly.

