# Supplementary material for: Molecular architecture of the 90S small subunit pre-ribosome
Source: eLife. 2017 Feb 28;6:e22086. doi: 10.7554/eLife.22086 (PMC5354517; doi:10.7554/eLife.22086)
Supplement: Supplementary file 1. — DOI: http://dx.doi.org/10.7554/eLife.22086.028 [file elife-22086-supp1.docx]

Table S1. Components and modeling of yeast 90S structure

| Components | Alias | Functional group | Residue number | Modeling remark | Key crosslinks for AFs or MS signals of r-proteins |
| --- | --- | --- | --- | --- | --- |
| 5' ETS |  |  | 700 | 462 nt modeled |  |
| 18S |  |  | 1800 | 1103 nt modeled |  |
| ITS1 |  |  | 361 | 12 nt modeled |  |
| U3 RNA |  |  | 333 | 157 nt modeled |  |
| Utp15 |  | 5' ETS factor, UTPA | 513 | WD domain (about 1-376): poly-A model (2YMU, residues 5-13 and 302-577)  CTD (about 377-513): de novo poly-A model | EMG1_2-UTP15_244  EMG1_2-UTP15_243  UTP15_341-UTP30_170 |
| Utp5 |  | 5' ETS factor, UTPA | 643 | WD domain (about 1-365): poly-A model (2YMU, residues 5-13 and 302-577)  CTD (about 366-553): de novo poly-A model | NOP1_168-UTP5_419 |
| Utp9 |  | 5' ETS factor, UTPA | 575 | CTD (about 381-518): de novo poly-A model |  |
| Utp8 |  | 5' ETS factor, UTPA | 713 | WD domain (about 1-402): poly-A model (2YMU, residues 5-13 and 302-577)  CTD (about 403-688): de novo poly-A model | UTP4_371-UTP8_700  UTP4_773-UTP8_700 |
| Utp4 |  | 5' ETS factor, UTPA | 776 | WD domain (about 1-776): poly-A model (2YMU, residues 9-577) | UTP18_418-UTP4_316  UTP10_644-UTP4_561  UTP4_371-UTP8_700  UTP4_773-UTP8_700  NOP58_93-UTP4_217 NOP1_229-UTP4_217 |
| Utp17 | Nan1 | 5' ETS factor, UTPA | 896 | WD domain (about 1-786): poly-A model (2YMU, 8-577)  Residues 807-848: poly-A model of ctUtp10-ctUtp17 complex (5WYL, Chen R. and Ye K. unpublished data) | NAN1_163-UTP10_644 |
| Utp10 |  | 5' ETS factor, UTPA | 1769 | N domain (residues 3-421): homology model of ctUtp10-ctUtp17 complex (5WYL, Chen R. and Ye K. unpublished data)  M domain (residues 463-808): homology model of ctUtp10 M domain (5WY3, Chen R. and Ye K. unpublished data)  C domain (about 809-1769): poly-A model based on two copies of importin Kap60 (2C1T) | UTP10_249-UTP18_219  UTP10_271-UTP21_245  UTP10_644-UTP4_561  NAN1_163-UTP10_644  UTP10_1754-UTP20_27  UTP10_1754-UTP20_36 |
| Utp1 | Pwp2 | 5' ETS factor, UTPB | 923 | WD domain (about 1-706): homology model (2YMU)  Residues 707-852: homology model of the ctUtp1 CTD (5ICA). | PWP2_27-UTP21_730  PWP2_27-UTP21_766  PWP2_85-UTP21_661  PWP2_85-UTP21_9  PWP2_123-UTP21_9  PWP2_129-UTP21_661  PWP2_129-UTP21_9 |
| Utp21 |  | 5' ETS factor, UTPB | 939 | Residues 20-671: Utp21 WD domain (4NSX).  Residues 793-939: homology model of ctUtp21 CTD (5ICA). | PWP2_27-UTP21_730  PWP2_27-UTP21_766  PWP2_85-UTP21_661  PWP2_85-UTP21_9  PWP2_123-UTP21_9  PWP2_129-UTP21_661  PWP2_129-UTP21_9  UTP10_271-UTP21_245  UTP18_219-UTP21_288  RPS16A_107-UTP21_533 |
| Utp12 | Dip2 | 5' ETS factor, UTPB | 943 | WD domain (about 1-685): homology model (2YMU)  Residues 747-913: homology model of the ctUtp1 CTD (5ICA). | DIP2_740-EMG1_213 |
| Utp13 |  | 5' ETS factor, UTPB | 817 | WD domain (about 13-644): homology model (2YMU)  Residues 660-810: homology model of the ctUtp1 CTD (5ICA) | RPS14A_2-UTP13_181  RPS14A_49-UTP13_555 |
| Utp18 |  | 5' ETS factor, UTPB | 594 | WD domain (235-591): homology model of the ctUtp18 WD domain (5IC7) | UTP18_219-UTP21_288  UTP10_249-UTP18_219  UTP18_418-UTP4_316 |
| Utp6 |  | 5' ETS factor, UTPB | 440 | Unassigned |  |
| Nop56 |  | 5' ETS factor, U3 snoRNP | 504 | Residues 8-417: homology model of Nop5 (3PLA) | NOP1_212-NOP56_164  NOP1_299-NOP56_32 |
| Nop58 |  | 5' ETS factor, U3 snoRNP | 511 | Residues 3-403: homology model of Nop5 (3PLA) | NOP58_93-UTP4_217 |
| Snu13 |  | 5' ETS factor, U3 snoRNP | 126 | Residues 5-126: yeast Snu13 (2ALE) |  |
| Nop1 |  | 5' ETS factor, U3 snoRNP | 327 | Residues 84-322: homology model of Fibrillarin (3PLA) | NOP1_212-NOP56_164  NOP1_299-NOP56_32  FCF1_35-NOP1_168  FCF1_35-NOP1_299  FCF1_133-NOP1_299  NOP1_229-UTP4_217 |
| Rrp9 |  | 5' ETS factor, U3 snoRNP | 573 | Residues 130-570: yeast Rrp9 WD domain (4J0X) | RPS4A_53-RRP9_114  RPS24A_11-RRP9_114  RPS24A_21-RRP9_122  RPS24A_49-RRP9_122  RPS4A_245-RRP9_350  RPS24A_11-RRP9_350  RPS9A_90-RRP9_430 |
| Bud21 |  | 5' ETS factor | 214 | Unassigned |  |
| Utp7 |  | 5' ETS factor | 554 | WD domain (about 1-333): poly-A model (2YMU, residues 5-13 and 302-577) | UTP22_745-UTP7_518 SOF1_449-UTP7_478  SOF1_449-UTP7_476 |
| Utp11 |  | 5' ETS factor | 250 | N-terminal α-helix (about 2-74): de novo poly-A model | BMS1_1075-UTP11_75  BMS1_1075-UTP11_71  RPS23A_70-UTP11_2  RPS23A_70-UTP11_3 |
| Mpp10 |  | 5' ETS factor | 593 | Residues 45-64: poly-A model of the Rpf2-Rrs1 complex (5WXL, Zheng S. and Ye K. unpublished data)  Residues 429-454: structure of Imp3-Mpp10 complex (5WXM, Zheng S. and Ye K. unpublished data) | IMP3_73-MPP10_392  IMP3_73-MPP10_459 |
| Imp3 |  | 5' ETS factor | 183 | Residues 29-161: structure of Imp3-Mpp10 complex (5WXM, Zheng S. and Ye K. unpublished data) | IMP3_73-RPS16A_140  IMP3_73-IMP4_63  IMP3_73-MPP10_459  IMP3_73-MPP10_392 |
| Imp4 |  | 5' ETS factor | 290 | Residues 84-267: homology model of Rpf2-Rrs1 complex (5WXL, Zheng S. and Ye K. unpublished data) | IMP3_73-IMP4_63 |
| Sas10 |  | 5' ETS factor | 610 | Unassigned |  |
| Sof1 |  | 5' ETS factor | 489 | WD domain (about 1-376): poly-A model (2YMU, residues 5-13 and 302-577) | RPS7A_148-SOF1_143  SOF1_449-UTP7_478  SOF1_449-UTP7_476 |
| Fcf2 |  | 5' ETS factor | 217 | Unassigned |  |
| Fcf1 | Utp24 | 5' ETS factor | 189 | Residues 57-182: homology model of Utp23 (4MJ7) | FCF1_35-NOP1_168  FCF1_35-NOP1_299  FCF1_93-RPS9A_92  FCF1_112-RPS22A_71 FCF1_133-NOP1_299 |
| Enp2 |  | 5' domain factor | 707 | WD domain (about 1-311): poly-A model (2YMU, residues 5-13 and 302-577) |  |
| Bfr2 |  | 5' domain factor | 534 | Unassigned |  |
| Lcp5 |  | 5' domain factor | 357 | Unassigned |  |
| Efg1 |  | 5' domain factor | 233 | Unassigned |  |
| Krr1 |  | Central domain factor | 316 | Residues 38-212: structure of the Krr1-Faf1 complex (4QMF) | KRR1_87-RPS14A_2  KRR1_102-RPS14A_92  KRR1_127-RPS14A_92  KRR1_245-RPS13_107  KRR1_245-RPS13_107 |
| Rrp5 |  | Central domain factor | 1729 | Residues 1408-1721: structure of the Rrp5 TPR domain (5WWM, Chen X. and Ye K. unpublished data). |  |
| Utp22 |  | Central domain factor | 1237 | Residues 81-1237: structure of the Utp22-Rrp7 complex (4M5D) | RPS1A_33-UTP22_732  RPS1A_219-UTP22_1007  UTP22_745-UTP7_518  RRP7_2-UTP22_1205  RRP7_292-UTP22_1018 |
| Rrp7 |  | Central domain factor | 297 | Residues 3-189: structure of the Utp22-Rrp7 complex (4M5D)  Residues 190-216: de novo poly-A model | RPS13_100-RRP7_255  RPS13_43-RRP7_255  RPS13_43-RRP7_244  RRP7_2-UTP22_1205  RRP7_292-UTP22_1018 |
| Rok1 |  | Central domain factor | 564 | Unassigned |  |
| Emg1 |  | 3' major domain factor | 252 | Residues 28-252: structure of yeast Emg1 dimer bound to substrate RNA (3OIN) | EMG1_177-RPS5_213  DIP2_740-EMG1_213  EMG1_2-UTP15_244  EMG1_2-UTP15_243 |
| Utp30 |  | Late factor | 274 | Residues 8-255: homology model of r-protein L1 (1MZP) | UTP15_341-UTP30_170 |
| Bms1 |  | Late factor | 1183 | Residues 33-284 and 787-1016: homology model of Tsr1 (5IW7, Wang B. and Ye K. unpublished data)  Residues 547-636: structure of the Rcl1-Bms1 complex (4CLQ) | BMS1_602-RCL1_46  BMS1_610-RCL1_46  BMS1_646-RCL1_213 |
| Rcl1 |  | Late factor | 367 | Residues 7-361: structure of the Rcl1-Bms1 complex (4CLQ) | BMS1_602-RCL1_46  BMS1_610-RCL1_46  BMS1_646-RCL1_213 |
| Kre33 |  | Late factor | 1056 | Unassigned |  |
| Nop14 |  | Late factor | 810 | Unassigned |  |
| Noc4 |  | Late factor | 552 | Unassigned |  |
| Utp20 |  | Late factor | 2493 | de novo poly-A model | UTP10_1754-UTP20_27  UTP10_1754-UTP20_36  RPS4A_198-UTP20_1596  RPS6A_131-UTP20_643  RPS8A_142-UTP20_1245  RPS11A_102-UTP20_1646  RPS24A_83-UTP20_27 |
| Utp14 |  | Late factor | 899 | Unassigned |  |
| Enp1 |  | Late factor | 483 | Residues 205-465: structure of Enp1 (5WWO, Zheng W. and Ye K. unpublished data) |  |
| Pno1 |  | Late factor | 274 | Residues 94-266: homology model of aDim2 (3AEV) |  |
| Rrp12 |  | Late factor | 1228 | Unassigned |  |
| Rrt12 |  | Late factor | 206 | Unassigned |  |
| Faf1 |  | Late factor | 346 | Unassigned |  |
| S0 | uS2 | 3' major domain | 252 | Unassigned | Weak MS signal. |
| S1 | eS1 | Central domain | 255 | Residues 20-233: yeast ribosome structure (4V88) | Strong MS signal. |
| S2 | uS5 | head-body junction | 254 | Unassigned | No MS signal. |
| S3 | uS3 | 3' major domain | 240 | Unassigned | Weak MS signal. |
| S4 | eS4 | 5' domain | 261 | Residues 9-245: yeast ribosome structure (4V88) | Strong MS signal. |
| S5 | uS7 | 3' major domain | 225 | Residues 20-225: yeast ribosome structure (4V88) | Strong MS signal. |
| S6 | eS6 | 5' domain | 236 | Residues 1-226: yeast ribosome structure (4V88) | Strong MS signal. |
| S7 | eS7 | Central domain | 190 | Residues 4-187: yeast ribosome structure (4V88) | Strong MS signal. |
| S8 | eS8 | 5' domain | 200 | Residues 2-200: yeast ribosome structure (4V88) | Strong MS signal. |
| S9 | uS4 | 5' domain | 197 | Residues 12-186: yeast ribosome structure (4V88) | Strong MS signal. |
| S10 | eS10 | 3' major domain | 105 | Unassigned | No MS signal. |
| S11 | uS17 | 5' domain | 156 | Residues 2-142: yeast ribosome structure (4V88) | Strong MS signal. |
| S12 | eS12 | 3' major domain | 143 | Residues 20-143: yeast ribosome structure (4V88) | Strong MS signal. |
| S13 | uS15 | Central domain | 151 | Residues 9-142: yeast ribosome structure (4V88) | Strong MS signal. |
| S14 | uS11 | Central domain | 137 | Residues 11-119: yeast ribosome structure (4V88) | Strong MS signal. |
| S15 | uS19 | 3' major domain | 142 | Unassigned | Weak MS signal. |
| S16 | uS9 | 3' major domain | 143 | Residues 3-127: yeast ribosome structure (4V88) | Strong MS signal. |
| S17 | eS17 | 3' major domain | 136 | Unassigned | Strong MS signal. |
| S18 | uS13 | 3' major domain | 146 | Unassigned | Strong MS signal. |
| S19 | eS19 | 3' major domain | 144 | Unassigned | Strong MS signal. |
| S20 | uS10 | 3' major domain | 121 | Unassigned | No MS signal. |
| S21 | eS21 | head-body junction | 87 | Unassigned | No MS signal. |
| S22 | uS8 | Central domain | 130 | Residues 2-130: yeast ribosome structure (4V88) | Strong MS signal. |
| S23 | uS12 | 5' domain | 145 | Residues 43-145: yeast ribosome structure (4V88) | Weak MS signal. |
| S24 | eS24 | 5' domain | 135 | Residues 2-102: yeast ribosome structure (4V88) | Strong MS signal. |
| S25 | eS25 | 3' major domain | 108 | Unassigned | Weak MS signal. |
| S26 | eS26 | Central domain | 119 | Unassigned | Weak MS signal. |
| S27 | eS27 | Central domain | 82 | Residues 4-82: yeast ribosome structure (4V88) | Weak MS signal. |
| S28 | eS28 | 3' major domain | 67 | Residues 5-67: yeast ribosome structure (4V88) | Strong MS signal. |
| S29 | uS14 | 3' major domain | 56 | Unassigned | No MS signal. |
| S30 | eS30 | 5' domain | 63 | Residues 23-52: yeast ribosome structure (4V88) | No MS signal. |
| S31 | eS31 | 3' major domain | 152 | Residues 102-152: yeast ribosome structure (4V88) | No MS signal. |
| Asc1 | RACK1 | 3' major domain | 319 | Unassigned | No MS signal. |

The table includes 51 AFs that are present in the mature 90S ([Zhang et al. 2016b](#_ENREF_74)) and all 33 r-proteins in 40S. The mass spectrometry (MS) signals for r-proteins in a reference state of 90S (ITS1-239/Noc4-TAP particle) are included. The PDB codes of the fitted structures and the template structures for poly-A or homology models are indicated.
