# Supplementary material for: Molecular architecture of the 90S small subunit pre-ribosome
Source: eLife. 2017 Feb 28;6:e22086. doi: 10.7554/eLife.22086 (PMC5354517; doi:10.7554/eLife.22086)
Supplement: Supplementary file 3. — DOI: http://dx.doi.org/10.7554/eLife.22086.030 [file elife-22086-supp3.docx]

Table S2. Statistics of data collection, structural refinement and model validation

|  | Noc4-TAP | ΔMtr4/Enp1-TAP | ΔDhr1/Enp1-TAP |
| --- | --- | --- | --- |
| **Data collection** |  |  |  |
| EM equipment | FEI Titan Krios | FEI Titan Krios | FEI Titan Krios |
| Voltage (kV) | 300 | 300 | 300 |
| Detector | Falcon II | Falcon III | Falcon III |
| Grid | Quantifoil 2/2 | Quantifoil 1.2/1.3 | Quantifoil 1.2/1.3 |
| Micrographs | 1769 | 1102 | 2055 |
| Particles for 3D classification | 127,198 | 195,317 | 420,755 |
| Pixel size (Å) | 1.42 | 1.76 | 1.76 |
| Defocus range (μm) | 1.5-5 | 1.5-3.5 | 2.0-4.0 |
| Electron dose (e^-^/Å^2^) | 40 | 40 | 50 |
| **Map refinement** |  |  |  |
| Particles for refinement | 12,643 | 73,543 | 30,995 (state 1) |
| Overall resolution of map (Å) | 8.7 | 4.5 | 8.7 |
| Map sharpening B-factor (Å^2^) |  | -135 | -629 |
| **Model composition** |  |  |  |
| Protein chains |  | 55 | 61 |
| Protein residues |  | 16547 | 19955 |
| RNA chains |  | 3 | 3 |
| RNA bases |  | 1619 | 1734 |
| **Structural refinement** |  |  |  |
| Map CC (whole unit cell) |  | 0.399 | 0.513 |
| Map CC (around atoms) |  | 0.491 | 0.682 |
| **Rms deviations** |  |  |  |
| Bonds (Å) |  | 0.0057 | 0.0052 |
| Angles (°) |  | 1.16 | 1.02 |
| **Validation (protein)** |  |  |  |
| Molprobity score |  | 1.96 (78^th^ percentile) | 1.94 (79^th^ percentile) |
| Clashscore |  | 8.23 (81^th^ percentile) | 8.61 (79^th^ percentile) |
| Good rotamers (%) |  | 95.99 | 96.07 |
| Ramachandran plot favored (%) |  | 91.16 | 92.23 |
| Ramachandran plot Outliers (%) |  | 0.27 | 0.25 |
| **Validation (RNA)** |  |  |  |
| Correct sugar puckers (%) |  | 98.52 | 98.44 |
| Good backbone conformations (%) |  | 74.61 | 77.87 |
